# Child Tobacco Smoke Exposure, Indoor Home Characteristics, and Housing Stability among a National Sample of U.S. Children

**DOI:** 10.3390/toxics10110639

**Published:** 2022-10-25

**Authors:** E. Melinda Mahabee-Gittens, Gang Han, Ashley L. Merianos

**Affiliations:** 1 Division of Emergency Medicine, Cincinnati Children’s Hospital Medical Center, College of Medicine, University of Cincinnati, 3333 Burnet Avenue, MLC 2008, Cincinnati, OH 45229, USA; 2 Department of Epidemiology and Biostatistics, Texas A&M University School of Public Health, College Station, TX 77843, USA; 3 School of Human Services, University of Cincinnati, P.O. Box 210068, Cincinnati, OH 45221, USA

**Keywords:** tobacco smoke exposure, secondhand smoke, thirdhand smoke, children, mold, pesticides

## Abstract

(1) Objectives: To examine the associations of child tobacco smoke exposure (TSE) with home quality and housing instability. (2) Methods. A secondary analysis of 32,066 U.S. 0–11-year-old children from the 2018–2019 National Survey of Children’s Health was conducted. Child home TSE status was defined as: no TSE: child lived with no smokers; thirdhand smoke (THS) exposure only: child lived with a smoker(s) who did not smoke indoors; and secondhand smoke (SHS) and THS exposure: child lived with a smoker(s) who smoked indoors. Home quality was assessed by the presence of mold or pesticide use and housing instability was assessed by home ownership, frequency of moves, and number of household members. We conducted weighted multivariable logistic regression and linear regression models while adjusting for important child covariates. (3) Results: In total, 12.3% had home THS exposure only and 1.6% had home SHS and THS exposure. Compared to children with no home TSE, children with home SHS and THS exposure were 2.60 times more likely (95%CI = 1.73, 3.92) to have mold inside their homes; 1.57 times more likely (95%CI = 1.09, 2.26) to live in homes where pesticides were used ≥1 time during the past 12-months; and more likely to have more frequent moves (β = 1.06, 95%CI = 0.62, 1.50). (4) Conclusions: Children with home TSE have higher rates of mold, pesticide use, frequent moves, and household members compared to children with no home TSE. Children with TSE should also be screened for home quality and housing instability and provided with appropriate interventions and resources.

## 1. Introduction

Although there is no safe level of tobacco smoke exposure (TSE) for children [1], almost one-in-four are exposed to tobacco smoke in their homes [2]. TSE rates are even higher in children who live in poverty, rented homes, and in neighborhoods in which there is litter, lack of greenspace, and homes in disrepair [2,3,4]. Children who live in poverty are typically housed in homes that are of lower quality in which they are more likely to be exposed to tobacco smoke, damp conditions, mold, and pests [5]. Indoor exposures such as these are concerning since children spend most of their time indoors [6]. Prior research indicates that damp conditions, water damage, mold, and pests such as cockroaches, are common environmental exposures in U.S. homes [7]. Indoor mold is more common in low-income homes that are older and that lack air conditioning and carpeting [8,9]. Pests such as cockroaches and the use of pesticides to combat infestations are also more common in lower income homes [8,9]. Children’s exposures to indoor TSE, mold, and cockroaches or other pests are associated with asthma, allergic rhinitis, upper respiratory infections, and other respiratory conditions [10,11].

Children living in poverty may also experience high rates of housing instability which may be manifest by frequent moves, overcrowding, and difficulties paying their rent [5]. Families who have high residential mobility rates may be forced to move due to evictions, housing foreclosures, or gentrification [12]. When children do not have stable housing or when there is overcrowding, the parent–child relationships that are essential to establish consistent routines, healthy behaviors, and active learning may be threatened [13]. Further, important ties to other family members, peers, school members, and community members, all of which support healthy child behaviors, may not be created or sustained [14]. For example, one study found that low-income families who had multiple moves or who were behind on their rent were more likely to report poor child and caregiver health and increased material hardships [15]. Further, other research indicates that children with unstable housing are at higher risk to have lower cognitive outcomes and adverse behavioral or mental health outcomes [5,14,16].

Since TSE rates, as well as housing quality and instability, are higher in children living in poverty, it is difficult to disentangle these factors. Moreover, there is a research gap in understanding the specific housing hardships that children with TSE encounter. However, such information is important to inform future holistic interventions and policies aimed at reducing children’s TSE and improving environmental factors that can ultimately improve their physical and mental health outcomes. Thus, the primary study objective was to assess the associations of TSE and housing characteristics. To accomplish this, we conducted a secondary analysis of a nationally representative sample of U.S. children who were 0–11 years of age. Household quality was assessed using measures of indoor mold and pesticide use and housing stability was assessed using measures of home ownership, the numbers of moves the child has had since birth, and the number of household members. Children’s TSE can consist of living with smokers who smoke inside the house; these children are exposed to both secondhand smoke (SHS) and thirdhand smoke (THS) or living with smokers who smoke outside of the house only; these children are exposed to THS only. Therefore, we compared the housing characteristics of children who did not live with smokers and had no home TSE to children with SHS and THS exposure and children with THS exposure only. We hypothesized that when compared to children with no home TSE, children with either SHS and THS exposure or THS exposure only would be more likely to have indoor mold and pesticide use in the past 12-months. We also posited that children with either SHS and THS exposure or THS exposure only would have an increased number of moves and household members.

## 2. Materials and Methods

### 2.1. Participants and Procedures

Data were obtained from parents and their 0–11-year-old children who were participants in the 2018–2019 National Survey of Children’s Health (NSCH). The NSCH is a cross-sectional survey that assesses the health and well-being of 0–17-year-old U.S. children conducted by the U.S. Census Bureau; detailed methodology on the 2018 and 2019 NSCH is published elsewhere [17,18]. The sample consisted of 32,066 children ages 0–11 years after excluding participants ages 12–17 years (*n* = 24,817), missing data on TSE (*n* = 609) and the outcome variables of interest including mold (*n* = 150), pesticides (*n* = 1652), and number of moves (*n* = 674).

### 2.2. Measures

#### 2.2.1. Child Home TSE Status

Caregivers reported whether their child lived with anyone who smoked combustible tobacco products (no, yes). If their response was yes, then caregivers reported whether the household smoker smoked inside the home (no, yes). The two questions were combined to create child home TSE, the independent variable of interest with three categories: (1) did not live with ≥1 smoker (i.e., no home TSE); (2) lived with ≥1 smoker who did not smoke inside the home (i.e., home THS exposure only); and (3) lived with ≥1 smoker who smoked inside the home (i.e., home SHS and THS exposure) [19,20].

#### 2.2.2. Mold inside the Home

Caregivers reported whether they had seen any mold, mildew, or other water damage signs on walls or other surfaces, with the exception of the shower and bathtub, inside their home during the past 12-months (no or yes) [19,20].

#### 2.2.3. Pesticide Use inside the Home

Caregivers reported whether they used pesticides to control for insects inside their home during the past 12-months (never or ≥1 time) [19,20].

#### 2.2.4. Household Characteristics

Caregivers reported: (1) how many times their child moved to a new address since their birth; and (2) how many people were living in their child’s household, which were both assessed continuously. Caregivers also reported: (3) their home ownership status (owned or rented) [19,20].

### 2.3. Covariates

The following sociodemographic covariates were included in this study: Child age, sex, race/ethnicity, emotional, developmental or behavioral problem; caregiver education level; and family household structure and federal poverty level. We included emotional, developmental, or behavioral problems since pesticide exposure has been associated with neurodevelopmental disorders among children [21].

### 2.4. Statistical Analysis

We used SPSS Complex Samples (version 28.0) for analysis and adhered to the 2018–2019 NSCH methodology guidelines, which included adjusting for sample weights that accounted for NSCH nonresponse, potential sampling frame issues, and to reflect the U.S. 0–11-year-old population [22,23]. Descriptive statistics were calculated for all variables and raw counts and weighted percentages are provided. A one-way ANOVA test for age and several chi-square tests for the categorical covariates (e.g., child sex) were conducted to assess differences based on the three home TSE groups. We conducted weighted multivariable logistic regression models to assess the associations between home TSE status and the three categorical outcome variables of mold inside the home, pesticide use inside the home, and home ownership status, while adjusting for the covariates. We present adjusted odds ratios (AORs) and 95% confidence intervals (CIs) for these models. Then, we conducted weighted multiple linear regression models to assess the associations between home TSE status and the two continuous outcome variables of the number of times the child has moved and the number of people living in the child’s home, while adjusting for the covariates. We present model coefficient β estimates and the corresponding 95%CIs for these models. All missing values were removed prior to analyses and *p* < 0.05 indicated significance.

## 3. Results

The average (SE) age of children was 5.7 years (0.04) and 48.7% were female (Table 1). Concerning race/ethnicity, 52.2% were non-Hispanic White, 24.3% were Hispanic, 12.5% were non-Hispanic Black, and 11.1% were non-Hispanic Other/Multiracial. Seven percent of children had an ongoing emotional, developmental, or behavioral problem. Over half (52.3%) of children’s caregivers obtained an education of college degree or higher, 64.7% lived with two parents who were currently married, and 39.7% and 32.3% were in the lowest and highest federal poverty levels, respectively.

### 3.1. Covariates and Child Home TSE Status

Concerning home TSE status, 86.4% of children had no home TSE, 12.3% had home THS exposure only, and 1.6% had home SHS and THS exposure. With the exception of child sex, all covariates differed based on home TSE status (see Table 1). Specifically, child mean age increased from the no home TSE to home SHS and THS exposure groups. A higher percentage of children with home THS exposure only were non-Hispanic White (58.8%) and higher percentages of children with home SHS and THS exposure were non-Hispanic White (58.8%) and Black (26.2%). Children with home THS exposure only and children with home SHS and THS exposure had higher percentages of parents who had a lower education of < high school graduate or equivalent (41.0% and 55.5%, respectively) compared to children with no home TSE (22.8%). Higher percentages of children with both home THS exposure only and home SHS and THS exposure had an emotional, developmental, or behavioral problem (10.5% and 16.7%, respectively), family structures other than two parents who were currently married, and lower federal poverty levels.

### 3.2. Child Home TSE Status and Mold inside the Home

A total of 11.0% (*n* = 3,117) of children had mold inside their homes during the past 12-months. Compared to children with no home TSE, children with home THS exposure only were 1.40 times more likely (95%CI = 1.12, 1.75) to have mold inside their homes during the past 12-months, while adjusting for the covariates (Table 2). Similarly, children with home SHS and THS exposure were 2.60 times more likely (95%CI = 1.73, 3.92) to have mold inside their homes compared to children with no home TSE. 

### 3.3. Child Home TSE Status and Pesticide Use inside the Home

A total of 47.8% (*n* = 13,993) of children lived in homes where pesticides were used ≥1 time during the past 12 months. Compared to children with no home TSE, children with home THS exposure only (AOR = 1.26, 95%CI = 1.09, 1.45) and children with home SHS and THS exposure (AOR = 1.57, 95%CI = 1.09, 2.26) were more likely to live in homes where pesticides were used ≥1 time during the past 12-months, while adjusting for the covariates (see Table 2).

### 3.4. Child Home TSE Status and Household Characteristics

Children moved an average of 1.3 times (0.02) since they were born and lived with 4.5 people (0.02). Compared to children with no home TSE, children with home THS exposure only (β = 0.34, 95%CI = 0.20, 0.47) and home SHS and THS exposure (β = 1.06, 95%CI = 0.62, 1.50) were more likely to move a higher number of times, while adjusting for the covariates (Table 3). Children with home THS exposure only (β = 0.13, 95%CI = 0.03, 0.24) were also more likely to live with a higher number of people compared to children with no home TSE, while adjusting for the covariates.

A total of 29.1% (*n* = 6245) of children lived in rented homes. No significant difference was found between child home TSE and home ownership statuses, while adjusting for the covariates (see Table 3). 

## 4. Discussion

This study demonstrates that in a nationally representative sample of 0–11-year-olds, children who were exposed to THS or who were exposed to both THS and SHS were at increased risk to live in homes that contained mold and at increased risk to live in homes where pesticides were used more frequently. It is well-established that children with TSE are at increased risk of short-term and long-term respiratory and infectious illnesses such as colds, ear infections, pneumonia, and asthma, due to the thousands of pollutants in SHS and THS [1]. Additional exposure to mold, pests, and pesticides increases children’s risk of asthma and allergic rhinitis, possibly due to microbial and antigen-related inflammatory responses [11]. In parallel with past research [2,4], we also found that children with TSE were at increased risk to live in poverty. Families with low-incomes are more likely to live in lower quality homes in which lack of heat or air conditioning and increased litter and other negative elements create fertile conditions for mold and pests and other environmental hazards which adversely affect children’s health [3,5,12]. Specifically, mold and damp home environments are present in approximately 18.7 million low-income homes [24]. Housing conditions present in low-income homes and public housing may facilitate the entry of mold spores and pests [24,25]. These factors include poor building designs and structural deficiencies; homes in disrepair; building openings in doorways, ceilings, windows, cracks, and HVAC systems; inadequate ventilation systems; and water leaks [24,26,27]. The combination of exposure to TSE pollutants plus exposure to mold and pesticides indicate the need for programs to screen children for TSE and these dangerous home exposures in order to identify children at risk for respiratory and allergic health risks that could potentially be lessened with interventions and home clean-up strategies [28].

This study also found that children with TSE were at increased risk to have more frequent moves and to live with more household members; both of these measures have been used to indicate housing instability [5]. Frequent moves during a child’s early life and unstable housing is associated with poor health and cognitive outcomes [15], possibly due to children’s inability to form meaningful and enduring connections with their family, peers, and school personnel [14]. Similarly, overcrowding, transitory housing, and concerns about rent payments can result in severed connections with parents and social supports and emotional strains [14]. Our results also indicate that children with THS exposure only were more likely to live with more household members, which is an important finding given past work suggesting that overcrowding and smaller spaces may result in increased exposure to THS pollutants [29]; however, future studies with validated measures of SHS and THS exposure are warranted.

The study’s strengths include the use of two waves of data from a sample of children with results that can be generalized to U.S. 0–11-year-olds. Limitations include the self-reported nature of the measures, which lacked biochemical validation of children’s TSE status, and objective assessments of the presence of home mold and use of pesticides. Future studies should incorporate objective biochemical measures of SHS and THS exposure and home inspections. Additionally, the current study focused on 0–11-year-olds and did not assess potential differences in TSE patterns in children based on their specific developmental stages. Future research should consider assessing these TSE patterns among children in specific age groups and stages of development, especially among those exposed to THS and exposed to SHS and THS. Finally, this study examined 2018–2019 NSCH data collected prior to the COVID-19 pandemic, and it is possible that housing characteristics and child TSE patterns changed as a result of the pandemic. Thus, these associations should be examined in later waves of NSCH or in studies that collected data after the pandemic.

In conclusion, we found higher rates of mold, pesticide use, frequent moves, and household members in children with home TSE compared to children with no home TSE. The results suggest that health practitioners should screen all children for TSE, as well as housing quality and instability, in order to connect families with resources such as free tobacco cessation medications from the state tobacco quitline and government assistance to help with housing issues.

## Figures and Tables

**Table 1:** Covariates Overall and Based on Child Home TSE Status among U.S. Children Ages 0–11 Years, 2018–2019 NSCH.

	Overall (N = 32,066)	Child Home TSE Status			
		No Home TSE (*n* = 27,720)	Home THS Exposure Only (*n* = 3929)	Home SHS and THS Exposure (*n* = 417)	*p*-Value
Characteristics	*n* (%) ^a^	*n* (%) ^a^	*n* (%) ^a^	*n* (%) ^a^	
**Child Age, M (SE)**	5.7 (0.04)	5.6 (0.04)	5.9 (0.12)	6.4 (0.28)	**0.003**
**Child Sex**					0.889
Male	16,734 (51.3)	14,479 (51.4)	2034 (50.6)	221 (50.7)	
Female	15,332 (48.7)	13,241 (48.6)	1895 (49.4)	196 (49.3)	
**Child Race/Ethnicity**					**<0.001**
Non-Hispanic White	22,263 (52.2)	19,143 (51.2)	2841 (58.8)	279 (58.8)	
Non-Hispanic Black	1908 (12.4)	1652 (12.5)	189 (9.7)	67 (26.2)	
Hispanic	3817 (24.3)	3367 (25.0)	429 (21.3)	21 (7.0)	
Non-Hispanic Other/Multiracial	4078 (11.1)	3558 (11.3)	470 (10.2)	50 (8.0)	
**Child Emotional, Developmental, or Behavioral Problem**					**<0.001**
No	29,426 (93.7)	25,641 (93.6)	3455 (89.5)	330 (83.3)	
Yes	2640 (7.0)	2079 (6.4)	474 (10.5)	87 (16.7)	
**Caregiver Education Level**					**<0.001**
High school graduate/GED or lower	4426 (25.5)	3184 (22.8)	1052 (41.0)	190 (55.5)	
Some college	7336 (22.2)	5661 (20.7)	1499 (31.0)	176 (35.5)	
College degree or higher	20,304 (52.3)	18,875 (56.5)	1378 (28.0)	51 (9.0)	
**Family Structure**					**<0.001**
Two parents, currently married	22,772 (64.7)	20,609 (67.6)	2037 (49.6)	126 (21.0)	
Two parents, not currently married	2307 (9.1)	1678 (8.3)	557 (13.8)	72 (16.6)	
Single parent	5365 (19.2)	4220 (17.7)	990 (27.4)	155 (39.3)	
Other/unknown family type	1622 (7.0)	1213 (6.4)	345 (9.2)	64 (23.1)	
**Federal Poverty Level**					**<0.001**
0–199%	8884 (39.7)	6880 (36.7)	1718 (55.1)	286 (80.4)	
200–299%	5280 (15.6)	4431 (15.6)	778 (16.3)	71 (11.3)	
300–399%	4849 (12.4)	4313 (12.9)	510 (10.1)	26 (3.6)	
≥400%	13,053 (32.3)	12,096 (34.8)	923 (18.5)	34 (4.7)	

Abbreviations: TSE, tobacco smoke exposure; THS, thirdhand smoke; SHS, secondhand smoke. ^a^ Raw counts and weighted column percents, unless noted otherwise.

**Table 2:** Child Home TSE Status and Mold and Pesticide Use the Inside Home among U.S. Children Ages 0–11 Years, 2018–2019 NSCH.

	Mold inside Home				Pesticide Use inside Home			
		Multivariable Logistic Regression ^b^				Multivariable Logistic Regression ^b^		
	*n* (%) ^a^	AOR	95% CI	*p*-Value	*n* (%) ^a^	AOR	95% CI	*p*-Value
**Child Home TSE Status**								
No Home TSE	2575 (10.3)	Ref	Ref	Ref	11,965 (47.3)	Ref	Ref	Ref
Home THS Exposure Only	449 (14.3)	**1.40**	**1.12, 1.75**	**0.004**	1728 (50.7)	**1.26**	**1.09, 1.45**	**0.002**
Home SHS and THS Exposure	93 (24.4)	**2.60**	**1.73, 3.92**	**<0.001**	240 (56.4)	**1.57**	**1.09, 2.26**	**0.015**
**Child Age**	-	1.00	0.98, 1.02	0.961	-	**1.03**	**1.02, 1.05**	**<0.001**
**Child Sex**								
Male	1606 (11.0)	Ref	Ref	Ref	7250 (48.2)	Ref	Ref	Ref
Female	1511 (10.9)	1.01	0.86, 1.18	0.901	6683 (47.4)	0.96	0.88, 1.05	0.358
**Child Race/Ethnicity**								
Non-Hispanic White	2014 (9.3)	Ref	Ref	Ref	9242 (44.5)	Ref	Ref	Ref
Non-Hispanic Black	239 (14.2)	**1.46**	**1.16, 1.86**	**0.002**	1102 (57.3)	**1.76**	**1.51, 2.07**	**<0.001**
Hispanic	448 (12.9)	**1.38**	**1.10, 1.72**	**0.005**	1833 (50.9)	**1.41**	**1.23, 1.62**	**<0.001**
Non-Hispanic Other/Multiracial	416 (11.2)	**1.27**	**1.02, 1.57**	**0.030**	1756 (46.2)	1.08	0.95, 1.22	0.256
**Child Emotional, Developmental, or Behavioral Problem**								
No	2,755 (10.7)	Ref	Ref	Ref	12,735 (47.8)	Ref	Ref	Ref
Yes	362 (14.9)	**1.36**	**1.05, 1.75**	**<0.001**	1198 (48.0)	0.92	0.79, 1.09	0.335
**Caregiver Education Level**								
High school graduate/GED or lower	468 (13.1)	1.01	0.81, 1.25	0.939	1957 (47.3)	**0.83**	**0.71, 0.96**	**0.013**
Some college	778 (12.0)	0.99	0.82, 1.19	0.875	3218 (47.3)	**0.88**	**0.78, 0.99**	**0.027**
College degree or higher	1871 (9.5)	Ref	Ref	Ref	8758 (48.3)	Ref	Ref	Ref
**Family Structure**								
Two parents, currently married	2122 (10.2)	Ref	Ref	Ref	9774 (47.6)	Ref	Ref	Ref
Two parents, not currently married	260 (11.1)	0.84	0.60, 1.16	0.291	945 (42.4)	**0.76**	**0.63, 0.93**	**0.007**
Single parent	602 (14.6)	1.13	0.92, 1.40	0.254	2439 (50.4)	1.01	0.89, 1.16	0.856
Other/unknown family type	133 (8.0)	**0.56**	**0.40, 0.79**	**<0.001**	775 (50.1)	1.02	0.83, 1.27	0.837
**Federal Poverty Level**								
0–199%	1089 (13.6)	**1.56**	**1.27, 1.91**	**<0.001**	4026 (48.1)	0.99	0.87, 1.13	0.918
200–299%	573 (10.8)	**1.33**	**1.07, 1.64**	**0.009**	2258 (47.9)	1.03	0.91, 1.18	0.638
300–399%	446 (10.9)	**1.40**	**1.11, 1.77**	**0.005**	2083 (48.5)	1.07	0.93, 1.22	0.333
≥400%	1009 (7.9)	Ref	Ref	Ref	5566 (47.2)	Ref	Ref	Ref

Abbreviations: TSE, tobacco smoke exposure; THS, thirdhand smoke; SHS, secondhand smoke; Ref, reference category. ^a^ Raw counts and weighted row percents. ^b^ Multivariable logistic regression models including all variables.

**Table 3:** Child Home TSE Status and Household Characteristics among U.S. Children Ages 0–11 Years, 2018–2019 NSCH.

	Number of Times Moved Since Born				Number of People Living in the Home				Home Ownership Status (Ref: Owns Home)			
		Multiple Linear Regression ^b^				Multiple Linear Regression ^b^				Multivariable Logistic Regression ^b^		
	M (SE)	β	95% CI	*p*-Value	M (SE)	β	95% CI	*p*-Value	*n* (%) ^a^	AOR	95% CI	*p*-Value
**Child Home TSE Status**												
No Home TSE	1.7 (0.05)	Ref	Ref	Ref	4.2 (0.04)	Ref	Ref	Ref	4896 (27.7)	Ref	Ref	Ref
Home THS Exposure Only	2.1 (0.08)	**0.34**	**0.20, 0.47**	**<0.001**	4.4 (0.06)	**0.13**	**0.03, 0.24**	**0.012**	1177 (36.3)	1.07	0.91, 1.27	0.414
Home SHS and THS Exposure	2.8 (0.23)	**1.06**	**0.62, 1.50**	**<0.001**	4.3 (0.15)	0.09	−0.21, 0.39	0.546	172 (47.5)	1.13	0.77, 1.67	0.522
**Child Age**	-				-	**0.03**	**0.02, 0.04**	**<0.001**	-	**0.97**	**0.95, 0.99**	**<0.001**
**Child Sex**												
Male	2.2 (0.09)	Ref	Ref	Ref	4.4 (0.06)	Ref	Ref	Ref	3307 (28.8)	Ref	Ref	Ref
Female	2.2 (0.09)	−0.01	0.08, 0.06	0.726	4.3 (0.06)	**0.06**	**0.01, 0.11**	**0.041**	2938 (29.4)	1.11	0.98, 1.26	0.093
**Child Race/Ethnicity**												
Non-Hispanic White	2.2 (0.09)	Ref	Ref	Ref	4.3 (0.06)	Ref	Ref	Ref	3114 (18.8)	Ref	Ref	Ref
Non-Hispanic Black	2.2 (0.10)	−0.02	−0.16, 0.12	0.820	4.3 (0.07)	−0.02	−0.12, 0.08	0.722	853 (50.7)	**2.76**	**2.30, 3.32**	**<0.001**
Hispanic	2.1 (0.10)	−0.06	−0.16, 0.04	0.250	4.4 (0.08)	**0.10**	**0.01, 0.18**	**0.032**	1239 (40.0)	**1.75**	**1.49, 2.06**	**<0.001**
Non-Hispanic Other/Multiracial	2.3 (0.09)	0.07	−0.01, 0.16	0.081	4.3 (0.07)	0.03	−0.04, 0.10	0.366	1039 (29.1)	**1.85**	**1.57, 2.19**	**<0.001**
**Child Emotional, Developmental, or Behavioral Problem**												
No	1.9 (0.08)	Ref	Ref	Ref	4.4 (0.06)	Ref	Ref	Ref	5537 (28.6)	Ref	Ref	Ref
Yes	2.5 (0.11)	**−0.65**	**−0.82, −0.48**	**<0.001**	4.3 (0.08)	0.12	−0.03, 0.26	0.109	708 (36.0)	1.20	0.97, 1.48	0.091
**Caregiver Education Level**												
High school graduate/GED or lower	2.1 (0.10)	0.01	−0.10, 0.12	0.892	4.4 (0.07)	0.05	−0.05, 0.15	0.327	1684 (46.6)	**1.75**	**1.47, 2.09**	**<0.001**
Some college	2.3 (0.10)	**0.14**	**0.04, 0.23**	**0.005**	4.3 (0.07)	−0.03	−0.11, 0.04	0.366	2166 (39.0)	**1.67**	**1.43, 1.96**	**<0.001**
College degree or higher	2.1 (0.09)	Ref	Ref	Ref	4.3 (0.07)	Ref	Ref	Ref	2395 (16.4)	Ref	Ref	Ref
**Family Structure**												
Two parents, currently married	1.9 (0.09)	Ref	Ref	Ref	4.6 (0.06)	Ref	Ref	Ref	3068 (21.1)	Ref	Ref	Ref
Two parents, not currently married	2.2 (0.11)	**0.35**	**0.20, 0.50**	**<0.001**	4.2 (0.07)	**−0.38**	**−0.49, −0.27**	**<0.001**	920 (51.7)	**2.05**	**1.66, 2.53**	**<0.001**
Single parent	2.3 (0.10)	**0.40**	**0.30, 0.50**	**<0.001**	4.0 (0.07)	**−0.56**	**−0.66, −0.47**	**<0.001**	1851 (43.6)	**1.37**	**1.16, 1.61**	**<0.001**
Other/unknown family type	2.4 (0.11)	**0.55**	**0.38, 0.72**	**<0.001**	4.4 (0.09)	**−0.21**	**−0.35, −0.06**	**0.005**	406 (33.5)	0.82	0.62, 1.08	0.153
**Federal Poverty Level**												
0–199%	2.3 (0.09)	**0.25**	**0.16, 0.34**	**<0.001**	4.7 (0.06)	**0.71**	**0.63, 0.78**	**<0.001**	3503 (49.3)	**4.20**	**3.49, 5.04**	**<0.001**
200–299%	2.2 (0.09)	**0.18**	**0.09, 0.28**	**<0.001**	4.4 (0.07)	**0.44**	**0.36, 0.52**	**<0.001**	1129 (25.7)	**2.03**	**1.66, 2.48**	**<0.001**
300–399%	2.1 (0.10)	0.05	−0.04, 0.14	0.306	4.2 (0.07)	**0.20**	**0.13, 0.27**	**<0.001**	630 (15.7)	**1.28**	**1.03, 1.61**	**0.029**
≥400%	2.1 (0.09)	Ref	Ref	Ref	4.0 (0.07)	Ref	Ref	Ref	983 (11.0)	Ref	Ref	Ref

Abbreviations: TSE, tobacco smoke exposure; THS, thirdhand smoke; SHS, secondhand smoke; Ref, reference category. ^a^ Raw counts and weighted row percents. ^b^ Multiple linear regression and multivariable logistic regression models including all variables.

## Data Availability

Data are publicly available on the NSCH website at https://www.childhealthdata.org/learn-about-the-nsch/NSCH (accessed on 24 January 2022).
